# Non-overlapping activities of ADF and cofilin-1 during the migration of metastatic breast tumor cells

**DOI:** 10.1186/1471-2121-14-45

**Published:** 2013-10-05

**Authors:** Lubna H Tahtamouni, Alisa E Shaw, Maram H Hasan, Salem R Yasin, James R Bamburg

**Affiliations:** 1 Department of Biology and Biotechnology, Faculty of Science, The Hashemite University, Zarqa 13115, Jordan; 2 Department of Biochemistry and Molecular Biology, Colorado State University, Fort Collins, CO 80523, USA

**Keywords:** ADF, Cofilin, Metastasis, Invadopodia, Adhesion, Lamellipodia

## Abstract

**Background:**

ADF/cofilin proteins are key modulators of actin dynamics in metastasis and invasion of cancer cells. Here we focused on the roles of ADF and cofilin-1 individually in the development of polarized migration of rat mammary adenocarcinoma (MTLn3) cells, which express nearly equal amounts of each protein. Small interference RNA (siRNA) technology was used to knockdown (KD) the expression of ADF and cofilin-1 independently.

**Results:**

Either ADF KD or cofilin KD caused cell elongation, a reduction in cell area, a decreased ability to form invadopodia, and a decreased percentage of polarized cells after 180 s of epidermal growth factor stimulation. Moreover, ADF KD or cofilin KD increased the rate of cell migration and the time of lamellipodia protrusion but through different mechanisms: lamellipodia protrude more frequently in ADF KD cells and are more persistent in cofilin KD cells. ADF KD cells showed a significant increase in F-actin aggregates, whereas cofilin KD cells showed a significant increase in prominent F-actin bundles and increased cell adhesion. Focal adhesion area and cell adhesion in cofilin KD cells were returned to control levels by expressing exogenous cofilin but not ADF. Return to control rates of cell migration in ADF KD cells was achieved by expression of exogenous ADF but not cofilin, whereas in cofilin KD cells, expression of cofilin efficiently rescued control migration rates.

**Conclusion:**

Although ADF and cofilin have many redundant functions, each of these isoforms has functional differences that affect F-actin structures, cell adhesion and lamellipodial dynamics, all of which are important determinants of cell migration.

## Background

Most eukaryotic cells sense motogenic signal gradients in their microenvironments and respond through cell polarization [1] and expand a single lamellipodium to establish directional migration [2]. Switching from the stationary state of the cell to the mobile state as in wound healing, gastrulation or metastasis depends on the actin cytoskeleton [3].

Migration and invasiveness of cancer cells is the hallmark of malignancy [4]. Cell migration is a highly integrated multistep process that includes development of cytoplasmic protrusions, attachment and traction [3]. The formation of these protrusions is driven by spatial and transient regulation of actin polymerization at the leading edge of polarized migratory cells [5]. Actin filament (F-actin) dynamics are regulated by actin binding proteins (ABPs) which are responsible for polymerization and treadmilling [6]. One of the most important families of ABPs is the ADF/cofilin (AC) family of proteins [7].

Vertebrates express three isoforms of ADF/cofilin encoded by three different genes: Actin Depolymerizing Factor (ADF), also known as destrin in mammals, non-muscle cofilin-1 (Cfl-1), and cofilin-2 (Cfl-2), which is enriched in muscle cells [8-10]. Human ADF and cofilin-1 are more than 70% identical in amino acid sequence [11]. At low concentrations with respect to actin subunits, ADF and cofilin-1 sever the filaments, but at higher concentrations they bind cooperatively to saturate F-actin and stabilize the severed fragments [9,12]. Also, ADF/cofilin depolymerize F-actin from the pointed end leading to enhancement of treadmilling [13]. ADF-actin has a much higher critical concentration for assembly than does cofilin-actin [11,14], and thus ADF but not cofilin can serve as a major monomer sequestering protein [15,16].

Metazoan ADF/cofilins are regulated by phosphorylation/dephosphorylation of a conserved serine (encoded ser 3 in human proteins) [17]. Known kinases are LIM Kinases LIMK1, LIMK2, and Testicular Kinases TESK1 and TESK2 [9]. The more specific cofilin phosphatases are chronophin and slingshot (SSH) [18-20]. AC proteins are pH-dependent in their interactions with F-actin [16,21-24].

Most research on ADF/cofilin proteins in metastatic invasion has focused on cofilin-1 (hereafter referred to as cofilin). Although ADF and cofilin can substitute for one another for many housekeeping activities in cultured cells [25], this is not the case during development. Cofilin null mice are not viable despite the fact that ADF is upregulated [26]. In contrast, ADF null mice are viable but show abnormal corneal thickening, suggesting that cofilin can rescue the lack of ADF except in corneal epithelial cells [27]. However, in ureteric bud (UB) epithelium, ADF and cofilin show considerable functional overlap, whereas simultaneous lack of both genes arrested branching morphogenesis at an early stage [10]. Likewise, most forms of ADF and cofilin from across phylogeny are able to compete similarly with myosin II for F-actin binding [28].

Silencing cofilin in colorectal cancer cells (Isreco1) did not interfere with their ability to undergo transwell migration across collagen in response to a chemotactic attractant. On the other hand, silencing of ADF, which represented only 17% of the total ADF/cofilin, significantly inhibited transwell migration, strongly suggesting different cellular functions of each protein in these cells [29].

Several studies have demonstrated an increase in cofilin amounts or in activity (dephosphorylated form) in cancer cells including cell lines derived from T-cell lymphoma (Jurkat) and carcinomas from the cervix (HeLa), colon (KM12), liver (HepG2) and kidney (COS1) [30], and in clinical tumor samples of oral squamous-cell carcinoma [31], renal cell carcinoma [32] and ovarian cancer [33]. In addition, overexpression of cofilin increases velocity of cell migration in *Dictyostelium*[34] and human glioblastoma cells [35]. Expression of wildtype or a non-phosphorylatable cofilin mutant in which ser 3 has been mutated to alanine (S3A) increases melanoma cell invasion [36].

However, opposite findings have also been reported. LIMK 1 activity, which should decrease active cofilin, is upregulated in invasive breast and prostate cancer cell lines and its overexpression increased motility of tumor cell lines [37,38]. Furthermore, suppression of LIMK2 in human fibrosarcoma cells or expression of a dominant negative LIMK1 in an animal model of tumor invasion, limited cell migration and efficiency to form dense colonies without affecting cell proliferation rate or viability [37,39,40]. Such opposite findings suggest that targets of LIMK1 and LIMK2, which include ADF as well as cofilin [9], bring about different effects, which could be dependent on relative amounts of ADF or cofilin that are expressed in the different tumor cell types.

MTLn3 mammary adenocarcinoma cells have been used extensively in the study of metastasis. In breast tumor microenvironments, gradients of EGF secreted by tumor associated macrophages (TAMs) act as chemo-attractants leading to cancer cell polarization toward EGF [41]. EGF binds to EGF receptor (EGFR) on the surface of MTLn3 cells leading to the activation of phospholipase Cγ (PLC-γ) and phosphatidylinositol 3-kinases (PI3K). ADF/cofilin are bound to phosphatidylinositol 4,5-bisphosphate (PIP_2_) in the plasma membrane of resting MTLn3 cells [42]. EGF-activated PLC-γ hydrolyzes PIP_2_ causing the release of ADF/cofilin from plasma membrane [43]. Active ADF/cofilin severs actin filaments creating new barbed ends that serve as nuclei for polymerization. New ATP-actin or ADP-Pi-actin subunits are preferred by the Arp2/3 complex, which is responsible for creating the branched actin filament arrays at the leading edge of migrating cells forming cell protrusions needed for crawling [44-47].

To study the roles of ADF and cofilin in cancer cell migration, we selected MTLn3 cells that expresses nearly identical amounts of each protein and silenced each in turn while performing a number of assays to assess the role of each in different aspects of polarized migration. Our results suggest that whereas many of the functions of cofilin and ADF are redundant, each of these isoforms has subtle functional differences that impact migratory cell behavior.

## Results

### Efficiency of infection of MTLn3

MTLn3 cells were infected for 72 h with adenoviruses for silencing ADF or cofilin, or for expression of fluorescent proteins. In double infection experiments, one of the viruses expressed GFP, whereas the other virus expressed mRFP. After 72 h, the cells were fixed, and scored for percentage of infection. In single infection experiments, 94.3% of the total cells expressed GFP while 96.8% expressed mRFP. In double infection experiments, 89.4% of the cells expressed GFP, 92.8% expressed mRFP and 86.9% expressed both GFP and mRFP. This demonstrates that the second virus infects in a cell autonomous manner (i.e. infection with the first virus neither enhances nor inhibits infection with the second virus).

### ADF and cofilin are equally expressed in MTLn3 rat adenocarcinoma cell lines

**Figure 1:**
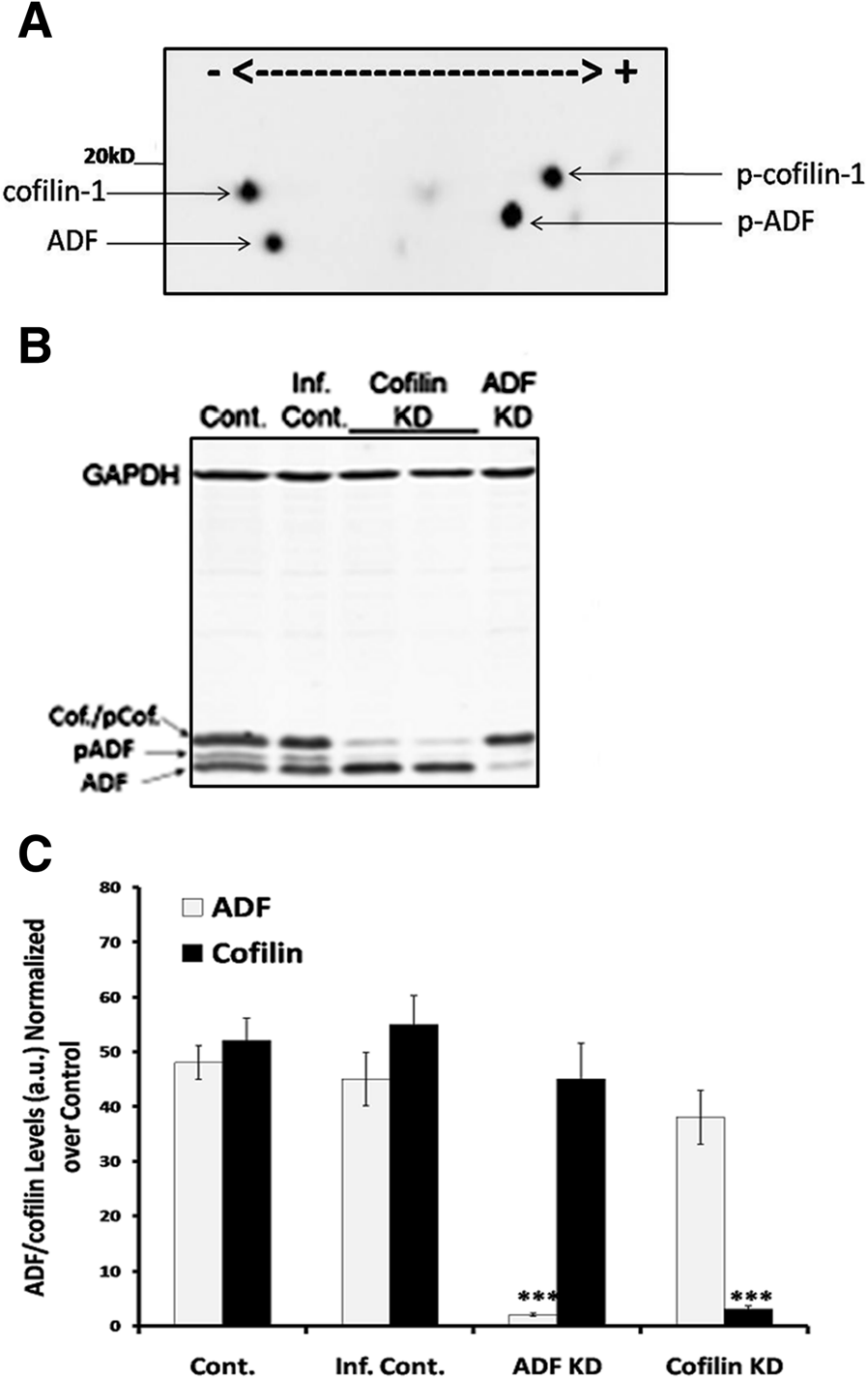
**ADF and cofilin expression levels in rat adenocarcinoma MTLn3 cells. A**. Representative 2D Western blot of extracted MTLn3 cell proteins immunolabeled with rabbit polyclonal antibody (1439) that recognizes ADF, phospho-ADF, cofilin, and phospho-cofilin with equal sensitivity. **B**. Representative Western blots of lysates from control uninfected MTLn3 cells, and cells infected for 72 h with adenovirus for expressing control siRNA, cofilin KD cells (duplicate culture extracts), and ADF KD cells. Blots were probed using the ADF/cofilin pan antibody rabbit 1439 and a monoclonal antibody to GAPDH as a loading control. The experiment was repeated three times and the corresponding quantification is shown in **(C)**. **C**. Quantification of ADF and cofilin levels in uninfected control, infected (human cofilin siRNA) control, ADF KD or cofilin KD cells. For the uninfected and infected controls the amounts of each shown are as a percent of the total ADF/cofilin. For the ADF KD and cofilin KD samples, the amounts are relative to the control values. *** p < 0.001 versus other treatments.

We examined the levels of cofilin and ADF (and their phosphorylated forms) in MTLn3 cell extracts by 2D Western blots using a polyclonal antibody that recognizes ADF and cofilin with equal sensitivity [48]. The lower ADF spots do not appear when blots are developed using the cofilin monoclonal antibody mAb22 (LHT, unpublished observations). MTLn3 cells express ADF and cofilin equally (Figure 1A, C), which prompted us to choose these cells to investigate the role(s) of ADF and cofilin during adhesion and migration.

### siRNA expression in MTLn3 cells results in an efficient and specific reduction of cofilin and ADF expression

To investigate the roles of ADF and cofilin in the invasive phenotype of MTLn3 cells, we used adenoviral mediated expression of hairpin RNAs to generate specific silencing siRNAs. Western blots of extracts from MTLn3 cells infected with adenovirus expressing either ADF or cofilin siRNA indicated that knock down (KD) of greater than 90% was obtained by 72 h post-infection (Figure 1B, C). ADF in ADF KD cells was reduced to 7% of controls [either uninfected (Cont.) or control virus infected (Inf. Cont.) cells] without effecting cofilin expression (Figure 1B, C). Similarly, cofilin in cofilin KD cells was reduced to 9% of controls without reducing ADF expression (Figure 1B, C). In the longer isocratic 15% acrylamide gels shown in Figure 1B, the phosphorylated ADF migrates above the ADF band and below the band containing cofilin and phospho-cofilin, which migrate together. ADF/cofilin levels in cells infected with adenovirus expressing a control non-silencing siRNA (Inf. Cont.) were not significantly different from uninfected controls (Figure 1B, C), demonstrating that adenovirus infection *per se* had no effect on ADF/cofilin expression. In all subsequent experiments, controls are cells infected with adenovirus expressing the non-silencing siRNA.

**Figure 2:**
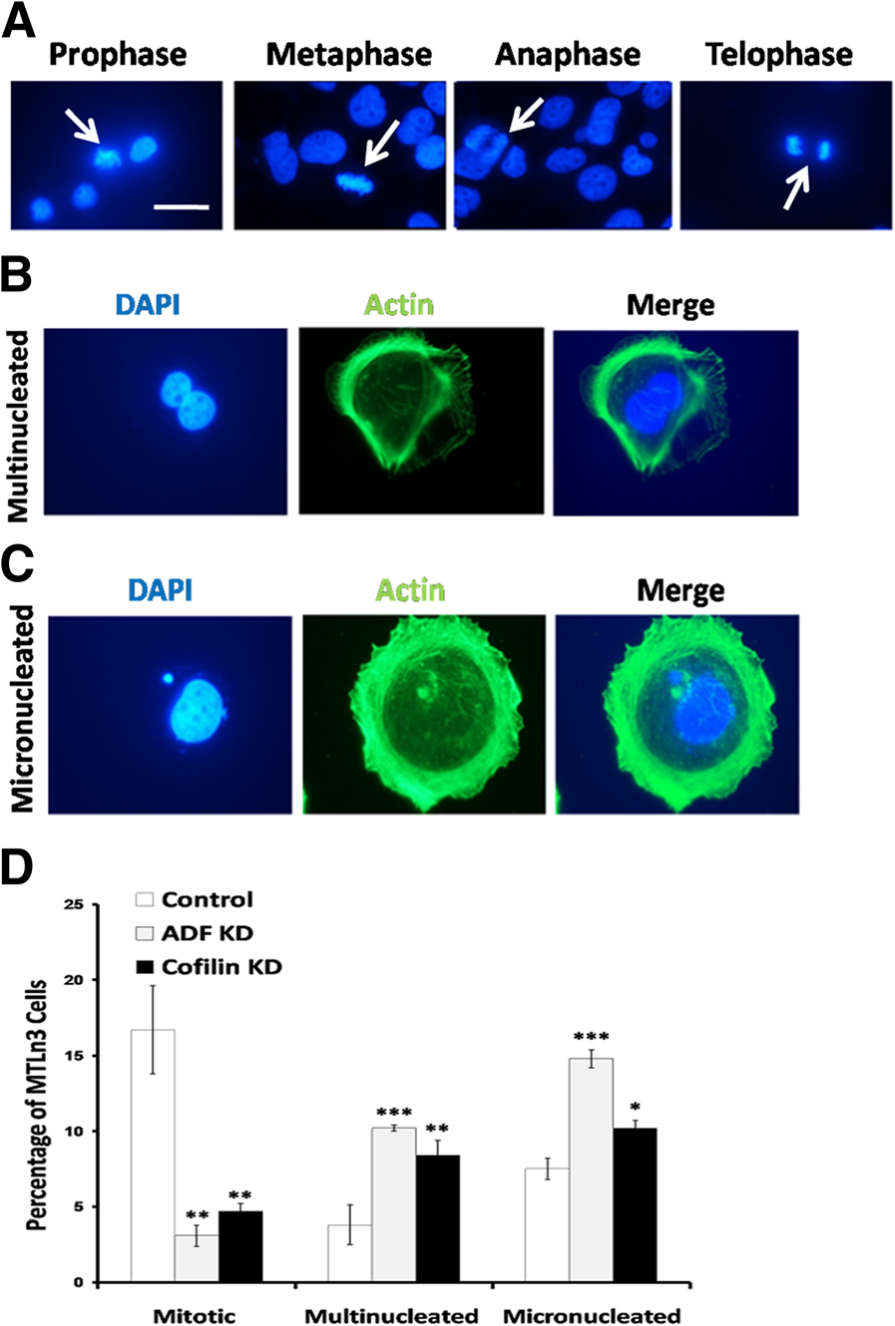
**ADF/cofilin depletion in MTLn3 cells decreases mitotic index, and increases multinucleation and micronuclei formation.** MTLn3 cells were stained with DAPI and fluorescent-phalloidin and three mitotic parameters were analyzed: mitosis **(A)**, multinucleation **(B)** and micronucleation **(C)**. **D**. Cells were scored as in **(A-C)** and mitotic index, percentage of multinucleation and micronucleation was calculated. n ≥ 600 cells in each experiment, three independent experiments. * p < 0.05, ** p < 0.01, *** p < 0.001 versus control. Scale bar: 10 μm.

Since proteins of the ADF/cofilin family have been shown previously to be involved in mitosis and cytokinesis [49], and to validate the adenoviral silencing of ADF and cofilin, we investigated certain mitotic parameters such as the mitotic index (no. of mitotic cells/total no. of cells × 100%) (Figure 2A, D), percentage of multinucleation (no. of cells having two or more nuclei/total no. of cells × 100%) (Figure 2B, D), and percentage of micronucleation (no. of cells having fragments or whole chromosomes lagging behind in anaphase/total no. of cells × 100%) (Figure 2C, D). As expected, the percentage of mitotic MTLn3 cells was decreased in siRNA-treated cells and both multinucleation and micronuclei formation increased as compared to the control infected cells (Figure 2D).

### ADF and cofilin silenced cells are characterized by an elongated shape and smaller cell area

To investigate the effect of ADF KD and cofilin KD on the morphology of MTLn3 cells, we measured cell length, width, the ratio of length to width (L/W ratio) and area of control and KD cells (Table 1). The cell length of ADF KD and cofilin KD cells increased significantly (p < 0.001) while the cell width decreased significantly (p < 0.001) when compared to the control cells. This in turn caused a significant increase in the L/W ratio (p < 0.001) and a significant decrease in cell area in ADF KD and cofilin KD cells (p < 0.001) when compared to control infected cells (Table 1).

**Table 1:** Suppression of ADF or cofilin causes cell elongation and area reduction

	**Length (μm)**	**Width (μm)**	**Length/width**	**Area (μm**^**2**^**)**
**Control**	40.4 ± 0.6	28.8 ± 0.9	1.4 ± 0.1	886.5 ± 3.8
**ADF KD**	60.1 ± 1.6^***^	11.9 ± 1.1^***^	5.2 ± 0.6^***^	515.9 ± 4.4^***^
**Cofilin KD**	75.1 ± 0.6^***^	9.0 ± 0.4^***^	8.3 ± 0.4^***^	535.9 ± 4.1^***^

Data are expressed as mean ± SEM, n ≥ 100 cells in each experiment, three independent experiments. ***p < 0.001 versus control.

### ADF and cofilin suppression affects MTLn3 cell polarization after EGF stimulation

**Figure 3:**
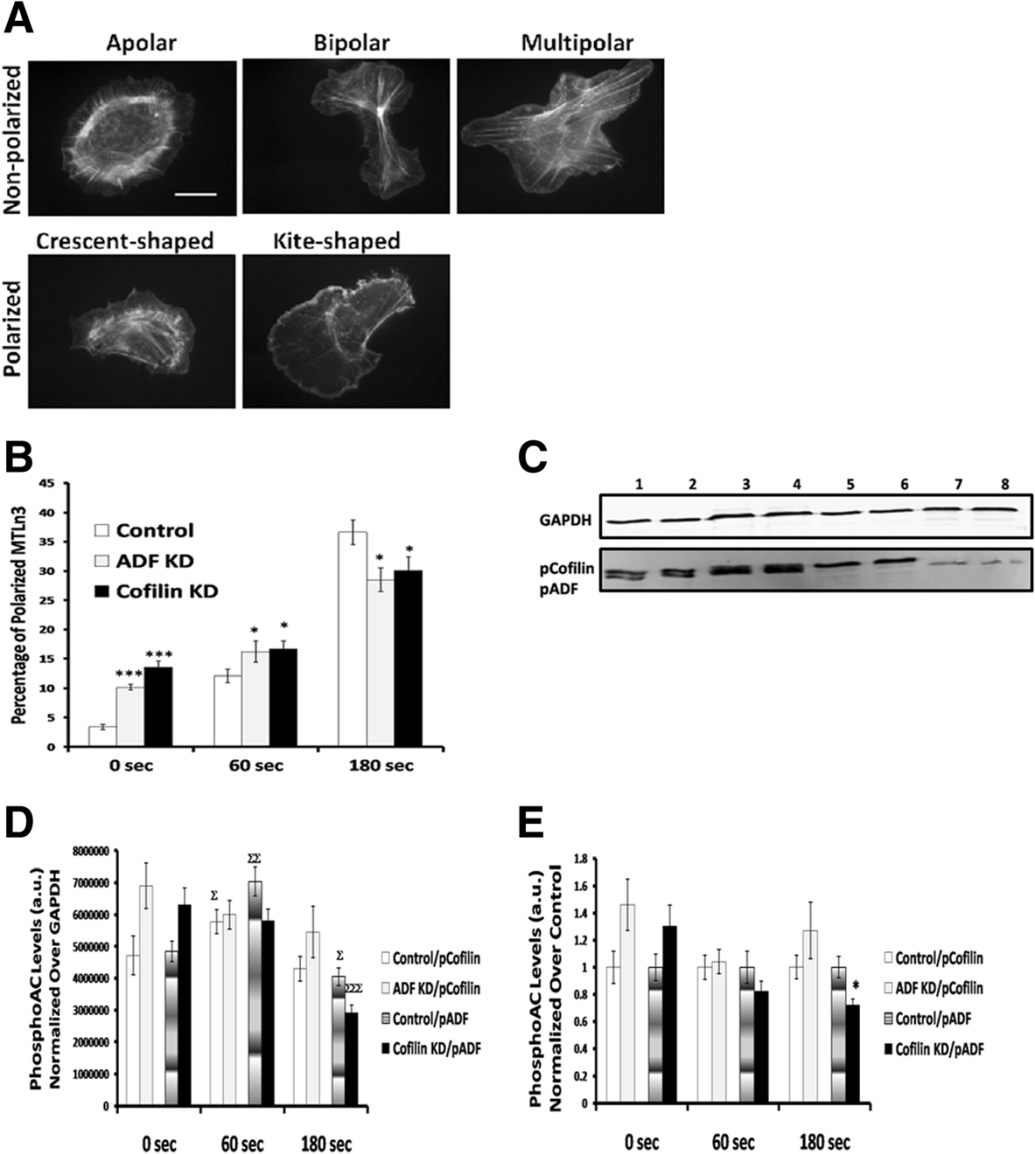
**ADF and cofilin suppression affects MTLn3 cell polarization and pADF levels after EGF stimulation. A**. MTLn3 cells were stimulated with 5 nM EGF for 0, 60 and 180 s and subdivided as polarized or non-polarized. **B**. MTLn3 cells were scored for percentage of polarized cells. ADF KD and cofilin KD cells are more polarized than control cells at the start of EGF treatment, but polarize less in response to EGF over the 180 s of EGF stimulation. **C**. Representative Western blot for pADF and pCofilin in control and EGF-treated MTLn3 cells after 180 s of EGF stimulation. Lane 1–2, control uninfected; lane 3–4 control infected; lane 5–6, ADF KD; Lane 7–8, cofilin KD. The experiment was repeated three times for 0, 60 and 180 s of EGF stimulation and the corresponding quantification is shown in **(D)**. **D**. Relative levels of pCofilin and pADF after 60 and 180 s of EGF stimulation compared to their levels of the same treatment at 0 s obtained from densitometry of immunoblots. **E**. Relative levels of pCofilin in ADF KD cells and pADF in cofilin KD cells compared to controls (set at 1) obtained from densitometry of immunoblots. * p < 0.05, *** p < 0.001 versus control at the same time point. ^∑^ p < 0.05, ^∑∑^ p < 0.01, ^∑∑∑^ p < 0.001 versus same treatment at 0 s. Scale bar: 10 μm.

To further analyze the impact of reducing ADF or cofilin expression on MTLn3 migratory morphology, control and KD cells were grown in starvation medium for 3 h and then were stimulated with 5 nM epidermal growth factor (EGF) for a period of 60 or 180 s, fixed, and stained with fluorescent-phalloidin. After imaging, cells were subdivided as having non-polarized or polarized morphology (Figure 3A). We compared the percentage of polarized cells in each period of time after EGF stimulation for control and treated MTLn3 cells (Figure 3B). ADF KD and cofilin KD cells showed a significant increase over controls in polarized morphology before EGF stimulation (p < 0.001) that was maintained over 60 s of EGF treatment (p < 0.05). However, by 180 s of EGF stimulation both ADF KD and cofilin KD cells showed a significant decrease in percentage of polarization as compared to control cells (p < 0.05) (Figure 3B). Thus, the ability of both ADF KD and cofilin KD cells to polarize in response to global EGF application is impaired.

For a more detailed analysis of the impacts of ADF and cofilin on cell shape, polarized cells were subcategorized into crescent- or kite-shaped, while non-polarized cells were subcategorized into apolar; bipolar or multipolar (Figure 3A) as described previously [50]. The percentage of cells in each category was scored in the control and KD cells (Table 2). The majority of the polarized control cells exhibited the crescent-shape morphology over the time period of EGF stimulation, whereas the kite shaped morphology was predominant in both ADF KD and cofilin KD cells prior to EGF addition. Polarized ADF KD and cofilin KD cells responded to EGF stimulation by rapidly (60 s) changing their shape from kite to crescent; however, polarized EGF-stimulated cofilin KD cells maintained a significantly higher percentage of kite-shaped cells over the entire time of EGF exposure, suggesting a decreased ability to release adhesions in their tail (Table 2). Most of the non-polarized cells in control and both KD cell types had the apolar shape even after EGF stimulation (Table 2).

**Table 2:** ADF and cofilin suppression affects MTLn3 cell polarization after EGF stimulation

**EGF stimulation**		**Subcategory**	**Control**	**ADF KD**	**Cofilin KD**
**0 sec**	Polarized	Crescent-shaped	68.3 ± 1.6	33.6 ± 1.2^***^	13.1 ± 0.6^***^
		Kite-shaped	31.7 ± 1.6	66.4 ± 1.2^***^	86.4 ± 0.6^***^
	Non-polarized	Apolar	91.3 ± 0.3	91.0 ± 0.1	71.5 ± 1.3
		Bipolar	8.7 ± 0.3	9.0 ± 0.1	9.7 ± 1.2
		Multipolar	0.0	0.0	18.8 ± 1.5
**60 sec**	Polarized	Crescent-shaped	75.0 ± 1.1	75.2 ± 1.9	49.3 ± 0.7^***^
		Kite-shaped	25.0 ± 1.1	24.8 ± 1.9	50.7 ± 0.7^***^
	Non-polarized	Apolar	91.4 ± 0.3	66.6 ± 0.2^***^	100.0^***^
		Bipolar	8.6 ± 0.3	34.4 ± 0.2^***^	0.0
		Multipolar	0.0	0.0	0.0
**180 sec**	Polarized	Crescent-shaped	79.8 ± 0.2	80.6 ± 0.4	48.5 ± 0.5^***^
		Kite-shaped	20.2 ± 0.2	19.4 ± 0.4	51.5 ± 0.5^***^
	Non-polarized	Apolar	91.2 ± 0.3	95.7 ± 0.3^***^	100.0^***^
		Bipolar	8.8 ± 0.3	4.3 ± 0.3^***^	0.0
		Multipolar	0.0	0.0	0.0

**Figure 4:**
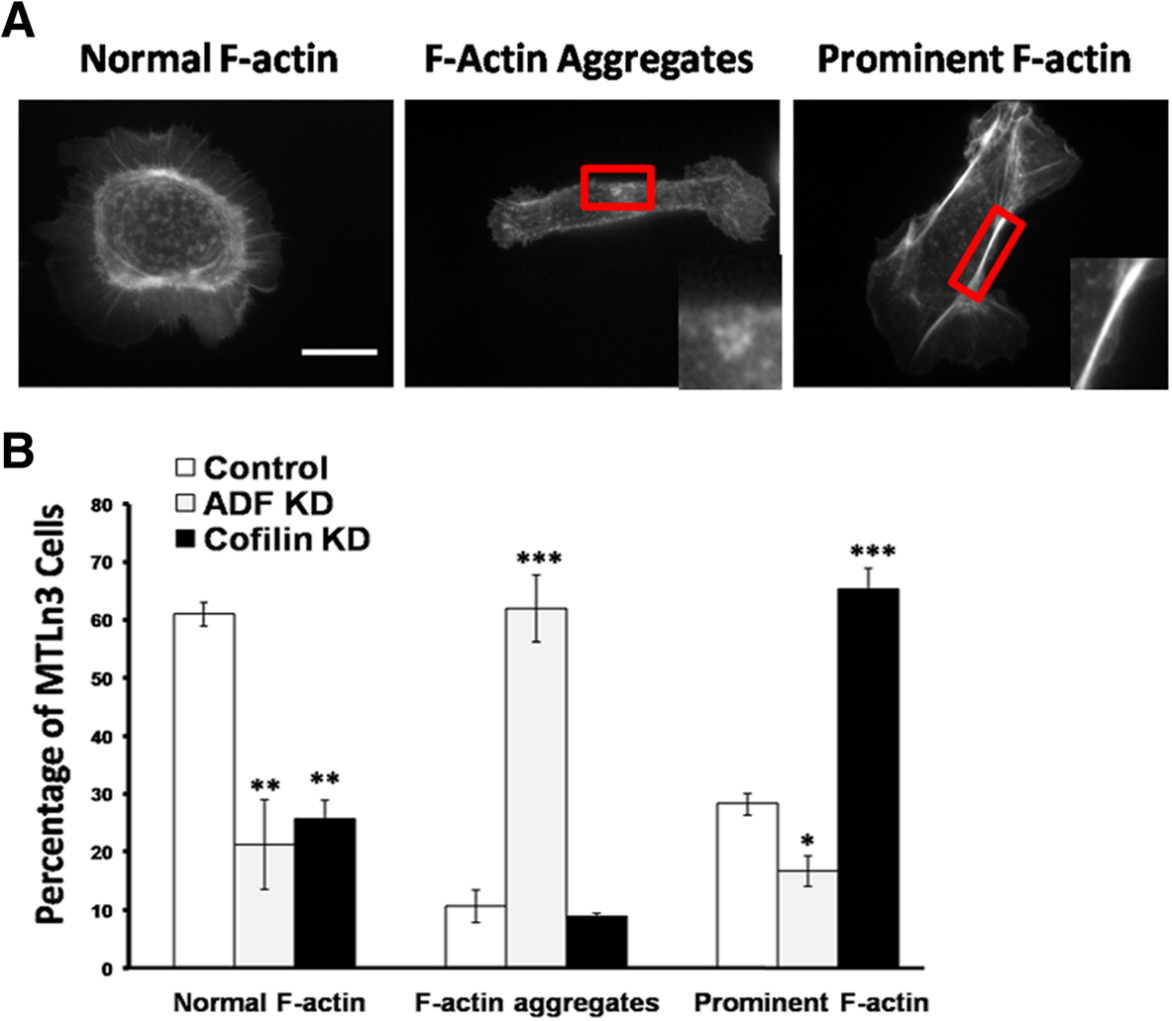
**Suppression of ADF or cofilin expression in MTLn3 cells causes changes in F-actin structure. A**. Cells were binned into three categories depending on their F-actin organization: normal F-actin, F-actin aggregates (magnified inset), and prominent F-actin (magnified inset) as stained with fluorescent-phalloidin. **B**. Cells were scored as in **(A)** and each type as a percentage of the total cells scored is shown. ADF KD cells showed enhanced F-actin aggregates while cofilin KD cells exhibited enhanced prominent F-actin. n ≥ 100 cells in each experiment, three independent experiments. * p < 0.05, ** p < 0.01, *** p < 0.001 versus control. Scale bar: 10 μm.

Polarized and non-polarized cells were subcategorized as in Figure 4A and the percentage of cells in each category was scored. Data are expressed as mean ± SEM, n ≥ 250 cells in each experiment, three independent experiments. ***p < 0.001 versus control.

### Changes in ADF and cofilin phosphorylation following EGF stimulation

The level of phospho-cofilin (pCofilin) in ADF KD cells and the level of pADF in cofilin KD cells (Figure 3D, E) were measured by western blotting after EGF stimulation (Figure 3C). Densitometry values of pCofilin and pADF at 60 and 180 s (normalized to GAPDH) where compared to the values at 0 sec of the same treatment. After 60 s of EGF stimulation, both pCofilin and pADF levels increased significantly in control cells, while pADF decreased significantly in both control and cofilin KD cells after 180 s of EGF stimulation (Figure 3D). In addition, the densitometry values from the blots were normalized to GAPDH and then expressed relative to pCofilin or pADF set at 1.0 in control cells. We found that pCofilin level did not change significantly in ADF KD cells during EGF stimulation as compared to pCofilin level in control cells, where as pADF levels decreased significantly (p < 0.05) by 180 s in cofilin KD cells (Figure 3E). The decline in pADF in cofilin KD cells is also evident in the blots of Figure 1B.

### ADF and cofilin KD cells exhibit changes in actin cytoskeleton

To determine if ADF KD and cofilin KD cells show changes in F-actin organization, MTLn3 cells were infected for 72 h, fixed and stained with fluorescent-phalloidin. Cells were observed and divided into three categories according to the phenotype of their actin cytoskeleton (Figure 4A) as described previously [50,51]. Both ADF KD cells and cofilin KD cells show significant (p < 0.01) decrease in normal F-actin (Figure 4B). However, ADF KD cells contain significantly more F-actin aggregates as compared to the control cells (p < 0.001), whereas cofilin KD cells showed a significant increase in prominent F-actin as compared to the control cells (p < 0.001) (Figure 4B).

### ADF and cofilin KD cells exhibit reduced ECM-degrading ability

**Figure 5:**
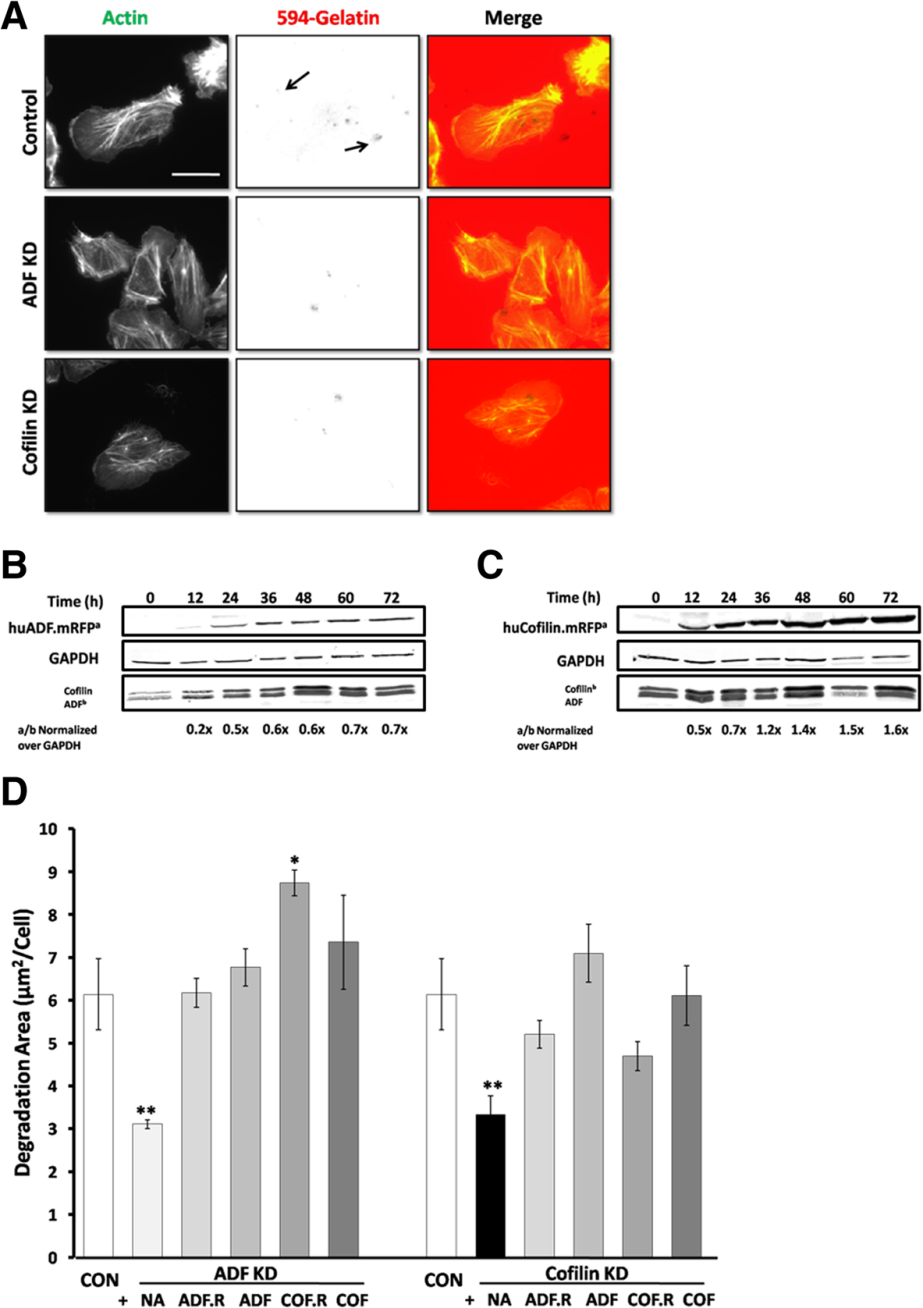
**Suppression of ADF or cofilin expression affects invadopodia formation. A**. Cells were cultured on fluorescent gelatin-coated cover slips and stained with fluorescent-phalloidin to visualize invadopodia (arrows). **B** and **C**. Representative Western blots for the expression of huADF.mRFP and huCofilin.mRFP in MTLn3 cells at 0, 24, 36, 48, 60 and 72 h after infection. Level of expression compared to endogenous ADF/cofilin was averaged from 3 or more blots. **D**. The degradation area of gelatin (μm^2^) was quantified in Metamorph and divided by number of cells in the same field and expressed as degradation area (μm^2^)/cell. n ≥ 30 cells in each experiment, three independent experiments. * p < 0.05, ** p < 0.01 versus control. Scale bar: 10 μm.NA, No Rescue Adenovirus; ADF.R, huADF.mRFP; ADF, huADF.RedTrack; COF.R; huCofilin.mRFP; COF, huCofilin.RedTrack.

To examine the ability of ADF KD or cofilin KD cells to degrade the extracellular matrix (ECM), control and silenced cells were cultured on Alexa 488- or Alexa 594-gelatin attached to a layer of cross-linked gelatin [52]. In this assay, proteolysis of the fluorescent-gelatin results in the appearance of dark non-fluorescent areas (Figure 5A). Both ADF KD and cofilin KD cells showed lower ECM degradation activity when compared to control infected cells (Figure 5D).

ADF or cofilin KD cells were co-infected with adenoviruses containing human ADF or cofilin cDNAs with conserved mutations that escaped siRNA silencing. Proteins were expressed as either the mRFP chimera huADF.mRFP (Figure 5B) or huCofilin.mRFP (Figure 5C), or as untagged versions huADF.RedTrack or huCofilin.RedTrack. Each of these viruses uses the CMV promoter to drive ADF/cofilin expression. The degradation area in these co-expressing cells was measured (Figure 5D). ADF KD cells expressing exogenous ADF (mRFP chimera or untagged) or cofilin (untagged) had a control-like degradation area (p > 0.05 versus control). Expressing exogenous huCofilin.mRFP in ADF KD cells increased the area of degradation when compared to control cells (p < 0.05). Degradation areas in cofilin KD cells expressing exogenous ADF or cofilin were somewhat more variable but were not significantly different from control (p > 0.05 versus control) (Figure 5D).

### Reduction of cofilin expression enhances cell adhesion to collagen I

**Figure 6:**
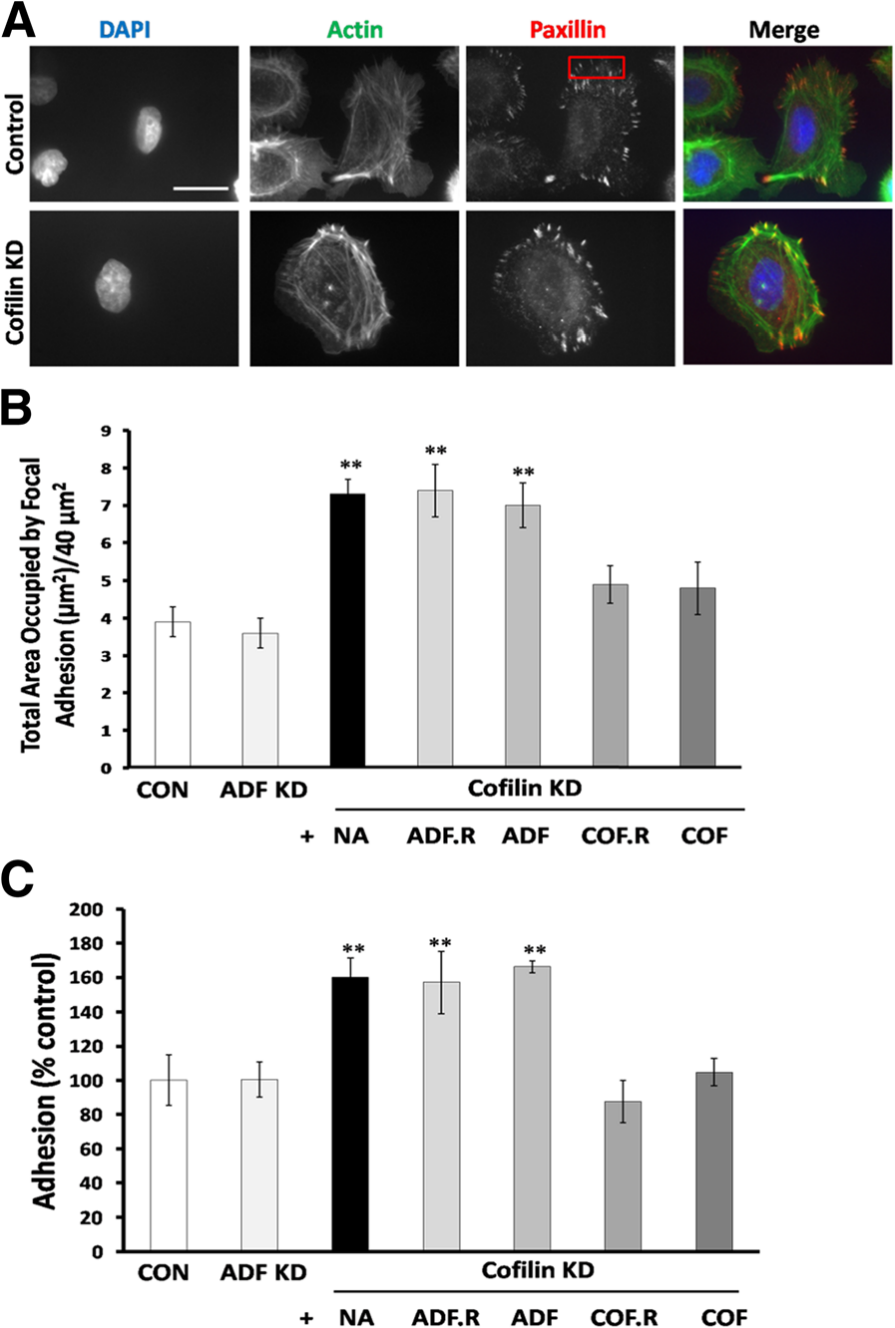
**Cofilin suppression enhances MTLn3 cell adhesion. A**. MTLn3 cells were stained for focal adhesion with mouse anti-paxillin primary antibody. The size and number of focal adhesions per unit area (40 μm^2^; red box) were measured in Metamorph, an average of 8 unit areas (boxes) at the leading edge of each cell were measured. **B**. Quantification of total area occupied by focal adhesion (μm^2^)/40 μm^2^ as: average number of focal adhesions/μm^2^ X average size of focal adhesions. n ≥ 25 cells in each experiment, three independent experiments. **C**. Cells were subjected to collagen I adhesion assay. Average values of cells adherent to plastic (not shown) were subtracted from average values adherent to collagen I. Average values of control cells were reported to 100%. Four independent experiments each performed in triplicate. ** p < 0.01 versus control. Scale bar: 10 μm.

Since ADF KD and cofilin KD cells showed changes in cell morphology and the actin cytoskeleton that suggested changes in cell adhesion, we next investigated the effect of ADF and cofilin depletion on MTLn3 cell adhesion. Cells were stained with anti-paxillin antibody and the size and number of focal adhesions were measured per unit area (40 μm^2^) at the leading edge of similarly shaped cells (Figure 6A), an average of 8 unit areas at the leading edge of each cell were selected. Cofilin silencing but not ADF silencing significantly increased the total area occupied by focal adhesions (μm^2^) per 40 μm^2^ area (7.3 ± 0.4 and 3.6 ± 0.4, respectively) as compared to control cells (3.9 ± 0.4) (Figure 6B).

Next, we infected cofilin KD cells with the different rescue adenoviruses expressing either ADF or cofilin and the total area occupied by focal adhesion/40 μm^2^ was measured in these co-expressing cells. Cofilin KD cells expressing exogenous cofilin (tagged or untagged) were not significantly different (p > 0.05) in cell adhesion (4.9 ± 0.5 and 4.8 ± 0.4, respectively) from control cells (Figure 6B), suggesting that the increased focal adhesion area arose from cofilin suppression. ADF expression, either as mRFP chimera or untagged, in cofilin KD cells did not restore focal adhesion area to the control level (7.4 ± 0.7 and 7.2 ± 0.6, respectively) (p < 0.05 versus control) (Figure 6B), demonstrating that ADF cannot substitute for cofilin in this process.

In addition, control, ADF KD and cofilin KD cells were seeded onto collagen I coated dishes, and adherent cells were quantified after 1 h (adhesion assay; [29]). We found that the number of adherent cells was greater in cofilin KD cells but not in ADF KD cells (159.9 ± 2.3% and 100.6 ± 3.5%, respectively), compared to control cells (100.0 ± 5.2%) (Figure 6C). Cofilin KD cells expressing exogenous huCofilin.mRFP or untagged cofilin, but not ADF, behaved like control infected cells (Figure 6C).

### Suppression of ADF or cofilin expression increases the rate of migration

**Figure 7:**
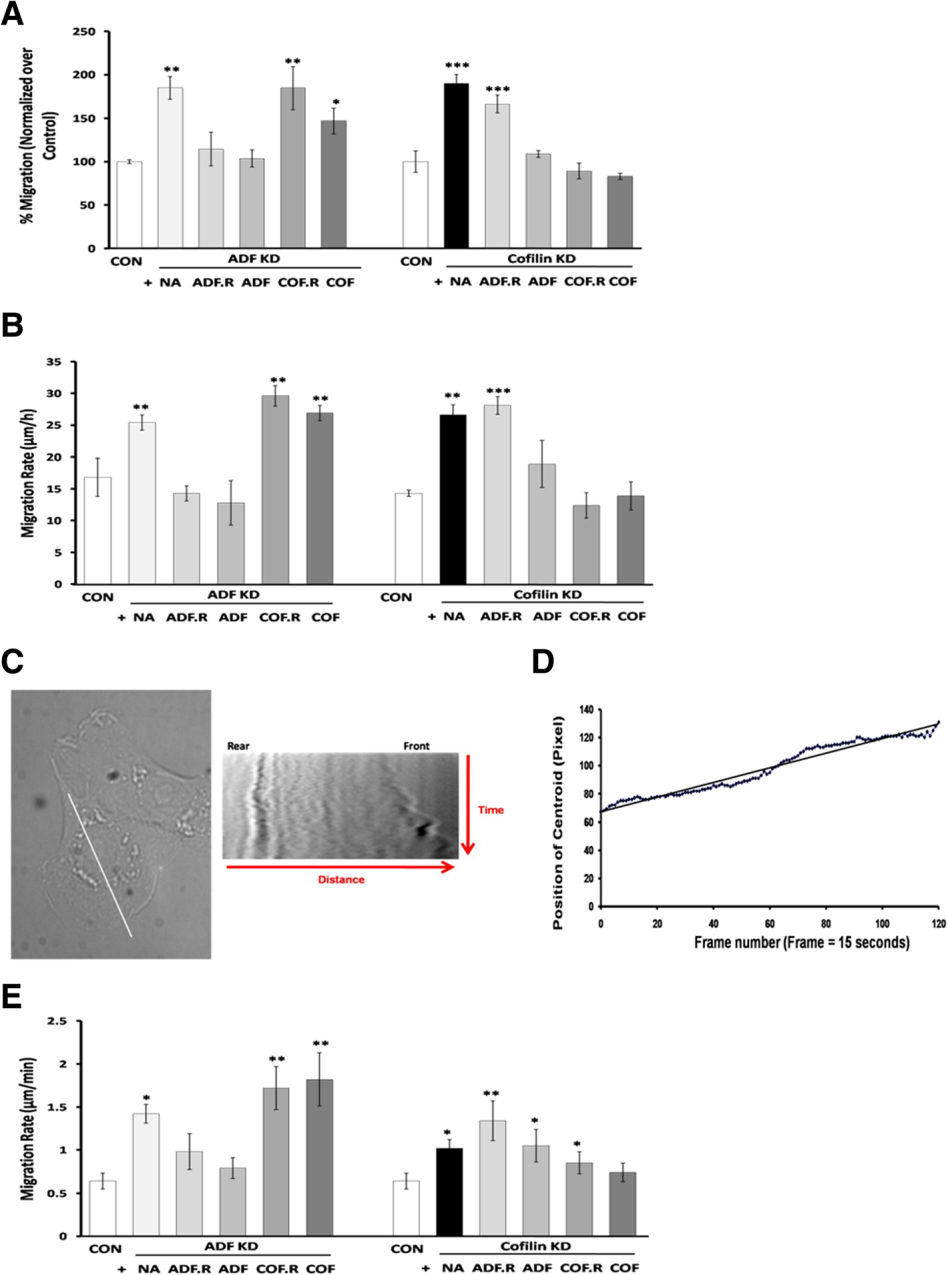
**Suppression of ADF or cofilin expression in MTLn3 cells enhances cell migration. A**. Cells were serum-starved, seeded on collagen I-precoated filters and then subjected to a migration assay. Cell migration is expressed as percent of control cells. Four independent experiments each performed in triplicate. **B**. MTLn3 cells were grown into monolayers and wounds were made with a sterile tip. The wound area was measured at 0 hr and 6 h, and migration rate was expressed as μm/h. Three independent experiments each performed in triplicate. **C**. The migration rate was measured from the cell centroid using kymography, four different regions of each cell were selected and a kymograph created for each region, only one of which is shown here. **D**. The kymograph was analyzed with a journal which tracks the front and rear membrane positions along the line at each time point and creates an average cell center position (centroid) which is then plotted versus time (frame number), from which a slope was calculated. **E**. The migration rate [slope (pixel/frame) × (1 μm/number of pixels) × (number of frames/number of min)] was averaged from 10 cells in each treatment, 4 measurements (slopes) per cell, three independent experiments. * p < 0.05, ** p < 0.01, *** p < 0.001 versus control.

Since ADF and cofilin depletion affected actin organization and cell polarization, we next analyzed the effect of knocking down ADF or cofilin on the migration of MTLn3 cells. We measured the number of ADF KD and cofilin KD cells migrating across type I collagen-coated filters (migration assay). Knocking down ADF or cofilin significantly (p < 0.01 and p < 0.001, respectively) enhanced MTLn3 cell migration by almost 80% compared to control cells (Figure 7A). Re-expressing exogenous ADF but neither tagged nor untagged cofilin in ADF KD cells decreased the number of cells migrating across collagen I-coated filters to the control level (Figure 7A). In cofilin KD cells, the number of migrating cells was reduced to control levels by expressing exogenous cofilin (Figure 7A). However, expressing exogenous untagged ADF but not ADF.mRFP, in cofilin KD cells also decreased the number of migrating cells (Figure 7A), suggesting that either the activity or accessibility of target binding by the chimeric huADF.RFP is less than that of the non-chimera.

The wound healing assay measures cell directed migration as a response to clearing of cells in a monolayer [53]. As expected from the results of the migration assay above, the migration rate of ADF KD and cofilin KD cells in a wound healing assay increased significantly when compared to the control (p < 0.01) (Figure 7B). The migration rate of ADF KD cells (25.4 μm/h ± 1.2) was reduced to that of control cells (16.8 ± 3.0 μm/h) upon expressing either exogenous huADF.RFP (14.3 ± 1.2 μm/h) or untagged ADF (12.8 ± 3.5 μm/h), p > 0.05 versus control, but not with expression of exogenous tagged or untagged cofilin (Figure 7B). For cofilin KD cells, re-expressing cofilin, tagged or untagged, restored the migration rate to that of control cells (12.4 ± 2.0 and 13.9 ± 2.2 μm/h, respectively). In addition, expressing exogenous untagged ADF but not ADF.mRFP in cofilin KD cells slowed them down (18.9 ± 3.7 μm/h) significantly (Figure 7B).

The migration rates of control and KD cells were measured by time lapse microscopy from the center position of the cell body (centroid) over 30 min using kymography. Four different line scans of each cell, each going through the centroid, were selected and a kymograph was created for each region (Figure 7C). The kymograph was then analyzed and the centroid position was plotted versus time and a slope was calculated (Figure 7D). The migration rate (μm/min) which equals [slope (pixel/frame) × (1 μm/number of pixels) × (number of frames/number of min)] was then calculated. Again, it was found that silencing either ADF or cofilin in MTLn3 cells significantly enhanced the cell migration rate (1.42 ± 0.11 and 1.02 ± 0.10 μm/min, respectively) as compared to control cells (0.64 ± 0.09 μm/min) (p < 0.05) (Figure 7E).

Expressing exogenous ADF (tagged or untagged), but not cofilin, in ADF KD cells reduced the migration rate to that of control cells (0.79 ± 0.12 μm/min) (p > 0.05 versus control) (Figure 7E). Expressing exogenous non-chimeric huCofilin in cofilin KD cells reduced the migration rate to that of the control cells (Figure 7E).

### ADF KD increases the time and frequency of protrusion and cofilin KD increases the time and persistence of protrusion

**Figure 8:**
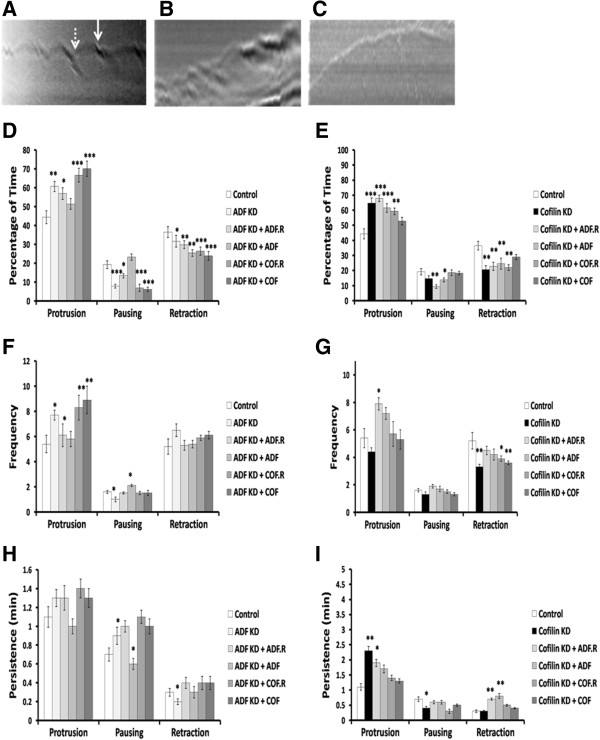
**Depletion of ADF or cofilin changes the lamellipodia history of migratory MTLn3 cells.** Representative kymographs of control **(A)**, ADF KD **(B)** and cofilin KD **(C)**. The lamellipodium history (protrusion, pausing, and retraction) of polar migratory MTLn3 was analyzed using kymograph images. The kymograph image was created from a line that crosses the cell centroid, and the reference point from which the lamellipodium is measured, is the position of the lamellipodium at time point zero. Arrow shows protrusion, dashed arrow shows retraction. **D** and **E.** The lamellipodia of ADF KD and cofilin KD cells spent the majority of their time (60.7% and 64.4%, respectively) protruding as compared to control cells (44.3%). This was rescued to a significant degree by re-expressing the untagged human ADF but not cofilin in ADF KD cells and human cofilin but not ADF in cofilin KD cells. **F** and **G**. The lamellipodia of ADF KD cells protruded significantly more frequently than in control and cofilin KD cells. Re-expressing of untagged human ADF in ADF KD cells restored the control phenotype. **H** and **I**. The protrusion of lamellipodia in cofilin KD cells was significantly more persistent than in control and ADF KD cells. Re-expressing huADF.RedTrack or huCofilin.mRFP or huCofilin.RedTrack restored the control phenotype in cofilin KD cells. n ≥ 10 cells in each treatment, three independent experiments, an average of 110 measurements. * p < 0.05, ** p < 0.01, *** p < 0.001 versus control.

The lamellipodial histories (protrusion, pausing, and retraction) of polar migrating MTLn3 cells were also analyzed using kymography (Figure 8A-C). Polar control cells spent less than half of the 30 min (44.3%) protruding and spent the rest of the time pausing (19.2%) or retracting (36.5%) (Figure 8D-E), and on average the lamellipodium fluctuated between protrusion and retraction 10 times per 30 min, while it paused less than two times over the same period (Figure 8F-G). On the other hand, polar ADF KD cells protruded 60.7%, paused 7.8% and retracted 31.6% of the 30 min (Figure 8D), and on average the lamellipodium fluctuated between protrusion and retraction 14 times per 30 min, while it paused once over the same period (Figure 8F). Polar cofilin KD cells protruded 64.8%, paused 14.6% and retracted 20.7% of the 30 min (Figure 8E), and on average the lamellipodium fluctuated between protrusion and retraction 8 times per 30 min, while it paused once over the same period (Figure 8G). The protrusion of the lamellipodium of cofilin KD cells persisted (2.3 min), significantly longer than in control (1.1 min) and ADF KD cells (1.3 min) (Figure 8H-I).

Expressing exogenous untagged ADF in ADF KD cells reduced the percentage of time ADF KD cells spend protruding (51.4%) by increasing the pausing time (23.2%) (Figure 8D). In addition, untagged ADF expression in ADF KD cells reduced the frequency of protrusion and increased pausing frequency (Figure 8F). Exogenous untagged cofilin decreased the percentage of time cofilin KD cells spend protruding (52.8%) and increased the percentage of pausing and retraction time (18.3% and 28.9%, respectively) (Figure 8E). In addition, both cofilin.mRFP and untagged cofilin expressed in cofilin KD cells decreased the protrusion persistence and increased the persistence of retraction (Figure 8I).

## Discussion

Most studies on MTLn3 mammary adenocarcinoma cells and many other tumor cell types that have addressed changes in the regulatory proteins that alter actin organization have focused on cofilin-1 [42,46,47,50], primarily because it was reported to be the major ADF/cofilin protein expressed in MTLn3 cells [54,55]. However, this determination used antibodies that had a much greater affinity toward cofilin than toward ADF. Using chick ADF as an antigen, we developed an antibody that has a strong affinity toward the epitope around amino acids 50–53 in chick ADF, which is conserved in both mammalian ADF and cofilin, and thus serves as a pan ADF/cofilin antibody in mammals [48]. Using this antibody we discovered that MTLn3 cells express nearly identical amounts of each isoform (Figure 1). Thus, these cells provided the ideal model system in which to determine if ADF and cofilin have fully redundant or overlapping roles in polarization and polarized migration.

In addition, since cofilin but not ADF is essential for normal cell behavior and its global inhibition would be detrimental to non-tumor tissue [26], demonstrating that ADF activity plays a different role in metastasis from cofilin might open up new avenues for therapeutic targeting. Thus, the aim of this study was to examine the requirements for ADF and cofilin individually for each step during polarization and migration of MTLn3 cells.

MTLn3 cells are large and flat making them suitable for quantitative imaging at the cellular and sub-cellular level. In addition, individual ADF or cofilin silencing did not significantly alter the expression of the other (Figure 1B, C), eliminating the need to study this compensatory mechanism which occurs in some cell types [28]. However, cofilin silencing did lead to increased amounts of the active (dephosphorylated) form of ADF (Figure 1B, Figure 3C), suggesting some compensation in this direction but the opposite (cofilin dephosphorylation in ADF KD cells) did not occur. The reason for this compensatory change in only one direction is likely due to the maintenance of greater F-actin pools in cofilin KD cells versus the actin aggregates that accumulate in ADF KD cells (Figure 4B). A major phosphatase involved in activating both ADF and cofilin is slingshot-1 L (SSH-1 L) which requires F-actin binding for its activity [18].

Dense aggregates of actin that stain with phalloidin have been observed in cultured cells in which both ADF and cofilin have been silenced [28]. Actin aggregate formation is blocked by the myosin II inhibitor blebbistatin [28], suggesting that the ability of ADF to compete with myosin II for F-actin binding leads to more aggregates in ADF KD than in cofilin KD cells as observed here (Figure 4). Previous studies showed that cofilin KD caused a significant actin reorganization represented by increased stress fibers compared to control MTLn3 cells [50]. In addition, siRNA suppression of cofilin in NIH3T3 and mouse neuroblastoma cells led to accumulation of F-actin and increase in the thickness of stress fibers [25]. Equally interesting are results from studies that expressed the kinase domain of LIMK [51], which showed enhanced actin aggregates. In this latter study ADF activity would be affected equally to cofilin. Although ADF is a more efficient monomer sequestering protein than cofilin [9,11], its major mechanism in blocking aggregate formation is probably through its competition with myosin II in the actomyosin contraction leading to aggregates. These differences between the two proteins activities led to different effects on actin cytoskeleton organization.

Focal adhesions are sites of large macromolecular assemblies containing integrins with linkages to cytoplasmic actin bundles [56,57], and collagen in the extracellular matrix [58]. We observed a significant increase in collagen I-mediated cell adhesion of cofilin KD cells and not ADF KD cells (Figure 6B, C). These findings imply that ADF and cofilin are not redundant in the MTLn3 cell attachment process. The enlarged focal adhesions certainly contribute to the accumulation of stress fibers in cofilin KD cells, producing a tension force by their contraction [59]. Such a force is needed for the forward movement of the cell body [60] but release from these adhesions is also needed for efficient movement.

Previous studies showed that LIMK knockdown suppressed fibronectin-mediated rat ascites hepatoma cell attachment and focal adhesion formation [61]. Furthermore, formation of focal adhesions in HeLa cells was substantially enhanced in cells transfected with a vector expressing the cofilin kinase TESK1 but was reduced in cells expressing a kinase inactive TESK1 which suppressed cofilin phosphorylation, as well as formation of stress fibers and focal adhesions in cells plated on fibronectin [62]. In addition, depletion of the actin-binding protein coronin 2A in MTLn3 cells led to a decreased rate of focal adhesion disassembly, which was mediated through increased phosphorylated cofilin; expression of an active mutant of cofilin (S3A) restored focal adhesion turnover to that of control cells [63]. In our work, the area occupied by focal adhesion in cofilin KD cells was restored to that of control cells when human cofilin but not ADF was re-expressed (Figure 6B, C). Taken together, these findings demonstrate that cofilin has a more prominent role than ADF in regulating cell adhesion, and thus in releasing tail focal adhesions necessary for the crescent cell morphology (Table 2).

Since ADF and cofilin are responsible for actin dynamics, and they are well-known regulators that trigger and maintain cell polarization [64], the significant decrease observed in the percentage of EGF-induced polarized cells in the ADF KD and cofilin KD cells compared to controls (Figure 3B) was expected. Overexpression in endogenously polarized chick embryo heart fibroblasts of a constitutively active mutant of LIMK or a pseudophosphorylated mutant of *Xenopus* ADF/cofilin in which ser 3 has been replaced by glu (S3E) caused the cells to lose their polarized phenotype and extend multiple lamellipodia [65]. Tail retraction of migrating polarized cells has been shown to require ADF/cofilin activity [66]. In ADF KD cells, the crescent shape is the dominant shape after EGF stimulation whereas tail persistence (kite-like morphology) is more prevalent in cofilin KD cells (Table 2) suggesting that cofilin is more responsible for tail retraction., These differences might arise because cofilin has a greater ability than ADF to reduce focal adhesion size (Figure 6) and/or because ADF has a somewhat greater ability to compete with myosin II for actin binding [28]; myosin II-mediated contractility also plays a role in tail retraction [60].

Our migration rate results are in agreement with those of others [50], who found that cofilin knockdown resulted in higher cell migration velocities and increased directionality. Cofilin KD MTLn3 cells followed a more linear path compared to the random walking path of control MTLn3 cells [50]. The higher migration rate observed in KD cells is consistent with our findings of lamellipodia history; ADF KD causes the cells to spend more time protruding and their protrusion is more *frequent* (number of times of protrusion/30 minutes) compared to control cells (Figure 8D, F). In addition, cofilin KD cells spend more time protruding because their protrusion is more *persistent* (average time of protrusion) compared to control cells (Figure 8E, G), probably due to enhanced adhesion of the protrusion.

Since ADF but not cofilin can serve as a major monomer sequestering protein [15,16], the effect of ADF KD on lamellipodia protrusion could be due to a greater alteration of the actin monomer pool than that obtained with cofilin siRNA treatment [11,15,67]. ADF depletion causes a decrease in G-actin. The availability and localization of G-actin monomer near the leading edge is essential for cell polarization and thus directional cell migration [29]. In addition, the spatiotemporal localization of G-actin regulates actin dynamics required for lamellipodia protrusion, and decreased G-/F-actin ratio at the leading edge has been found to be associated with pausing and retraction of protrusions [68].

## Conclusion

In conclusion, we have demonstrated that although both ADF and cofilin are redundant for many cell behaviors, there are subtle differences in how these proteins affect cell adhesion and migration that are likely to be important in understanding the migration of different metastatic tumor cells. It should be pointed out that our analysis has been restricted to migration on a two dimensional substrate. Cells traversing through a 3 dimensional network in which adhesions are not formed in a distinct plane may show additional differences in behavior depending on their relative amounts and activities of ADF and cofilin [69].

## Methods

### Cell culture

MTLn3 rat mammary adenocarcinoma cells were a generous gift from Dr. Maryse Bailly, UCL Institute of Ophthalmology, London. MTLn3 cells were cultured in α-modified Eagle’s medium (α-MEM) (GIBCO, Invitrogen, USA), supplemented with 5% fetal bovine serum (FBS) (Euroclone, Italy), 5% glutamine (Lonza, Belgium), and 1% Ampicillin/Streptomycin (PAA, Austria) at 37°C in a humidified 5% CO_2_ incubator. MTLn3 cells were infected with adenovirus at a multiplicity of infection (MOI) of 25 and all experiments were performed 72 h post infection. For EGF stimulation, MTLn3 cells were washed twice with sterile PBS, and then grown in starvation medium [0.35% bovine serum albumin (BSA); BioBasic Inc., Canada] for 3 h at 37°C. EGF (Invitrogen) (5 nM) in starvation medium was added to the cells for 60 or 180 s [43,46].

### Design of silencing vectors and infection procedure

Vectors for expressing small interfering RNAs for rat ADF and cofilin were made by inserting DNA oligonucleotides in a plasmid expression vector (pSuper) [70] containing the H1 polymerase III promoter. Modified inserts including the H1 polymerase III promoter from the pSuper vector were excised and ligated into pShuttle and/or pAdTrack vectors [71]. The DNA oligos (Ambion Applied Biosystems, USA) used to generate the siRNA for rat cofilin was 5′-AAGGTGTTCAATGACATGAAA-3′ [42] whereas that for rat ADF was 5′-AAGTGATTGCAATCCGTGTAT-3′ [72]. The oligonucleotide for generating human cofilin siRNA, used as a control in the MTLn3 cells, was 5′-AAGTCTTCAACGCCAGAGGAG-3′. The pShuttle and pAdTrack plasmids containing the DNA to generate hairpin RNAs were then used to make adenovirus as described previously [73].

### Western blot analysis

Cells were lysed in cold lysis buffer (2% SDS, 10 mM Tris pH 7.5, 10 mM NaF, 2 mM EGTA, 10 mM dithiothreitol). Cell extracts were heated in a boiling water bath for 5 min and sonicated. Aliquots of lysates were diluted in 4× SDS-PAGE sample buffer (0.5 M Tris–HCl pH 6.8, 2% SDS, 20% glycerol, 20% 2-mercaptoethanol and 0.16% bromophenol blue) and proteins resolved by electrophoresis on 15% SDS-polyacrylamide gels. Proteins were transferred onto nitrocellulose membranes and were blocked using 1% (w/v) BSA in Tris-buffered saline (TBS), and exposed overnight at 4°C to the primary antibodies [mouse mRFP (1:1000; Abcam, USA), mouse GAPDH (1:6000; CHEMICON, USA), rabbit polyclonal antibody (1439) that recognizes cofilin, phospho-cofilin, ADF and phospho-ADF with equal sensitivity (1:2000) [48], rabbit polyclonal (4321) that recognizes phospho-cofilin and phospho-ADF with equal sensitivity (1:2000) and mouse cofilin (mAb22) (1:250) [48] diluted in 1% BSA in TBS containing 0.05% Tween 20. After washing and incubation with appropriate secondary antibodies conjugated to Alexa680 or Alexa800, stained bands were imaged using the LiCor Odyssey Infrared Imaging System. Signals were quantified using TotalLab software (Nonlinear Dynamics).

For two dimensional Western blots, proteins in cell extracts were precipitated with chloroform/methanol [74] and the protein pellet was rehydrated in 8 M urea, 2% IGEPAL, 18 mM dithiothreitol. Proteins were separated on a precast pH 3–10 gel according to the manufacturer’s protocol (IPGphor Isoelectric Focusing System), followed by SDS-PAGE on 15% isocratic gels and then transferred onto nitrocellulose membrane. ADF and cofilin proteins were detected with the 1439 rabbit antibody.

### Cell staining and microscopy

MTLn3 cells were plated on sterile glass cover slips pre-coated with collagen I. Briefly, cover slips were coated with ice-cold freshly prepared collagen I (10 μg/ml) [for 1 ml: 100 μl 10× cold PBS, 25.5 μl 1 N NaOH, 873.4 μl distilled H_2_O, 1.1 μl of 9.03 mg/ml collagen I (Sigma Aldrich)]. Each cover slip was treated with 150 μl collagen I, left for 1 h at 37°C and then washed three times with PBS. Cells were fixed with 4% paraformaldehyde (Sigma Aldrich) in cytoskeleton buffer with sucrose (CBS) [10 mM MES, pH 6.1, 138 mM KCl, 3 mM MgCl_2_, 10 mM EGTA, 0.32 M sucrose] for 45 min (this and subsequent steps are at room temperature). Cells were then washed three times five min each with 0.1% Triton X-100 in PBS. F-actin was stained with fluorescent-conjugated phalloidin (Invitrogen) in CBS for 1 h. To visualize adhesion structures, cells were incubated with anti-paxillin antibody (BD Pharmingen, USA) (1:250 dilution in 2% BSA in CBS) for 1 h and then with fluorescent-conjugated goat anti-mouse IgG (1:400 dilution in 2% BSA in CBS) for 1 h. Cells were then mounted with Prolong Gold Antifade containing DAPI (Invitrogen). Images were captured using either a 20× NA 0.75 or 60× NA 1.4 objectives on an inverted Nikon microscope with a CCD camera and operated by Metamorph software (Molecular Devices). For time lapse, cells were plated onto glass-bottom dishes and infected for 72 h. Cells were then washed twice with α-MEM, starved for 3–4 h and imaged at 4 frames per min for 30 min in a bath application of 5 nM EGF [50], using Olympus confocal microscope equipped with a 37°C stage and 5% CO_2._

### Adhesion assay

Seventy-two hours after infection, cells were suspended in α-MEM-0.35% BSA and replated onto 10 μg/ml collagen I-precoated 96-well culture dishes at the concentration of 5 × 10^4^ cells/well. After incubation for 1 h at 37°C, cells were washed twice with PBS, and adherent cells were fixed in 4% paraformaldehyde for 30 min and stained with 1% borax and 1% methylene blue. After solubilization with 1% SDS, absorbance (630 nm) was measured [29].

### Invadopodia assay

Ethanol-flamed sterile 18 mm glass cover slips were placed in the wells of a 12-well tissue-culture plate and were coated with 50 μg/ml poly-D-lysine for 20 min at room temperature. The coverslips were then covered with 0.5% glutaraldehyde for 15 min, and then were coated with 37°C-preheated 0.2% gelatin (Sigma-Aldrich) and Alexa Fluor 488 or 568-gelatin (Invitrogen) mixture at a 8:1 ratio for 10 min at room temperature. The residual reactive groups in the gelatin matrix were quenched with 5 mg/ml sodium borohydride for 15 min at room temperature. Cells were plated at a concentration of 2 × 10^4^/cover slip and incubated at 37°C for 12 h. Cells were stained for F-actin with fluorescent phalloidin [52].

### Migration assay

Adenovirus-infected cells (1×10^6^) were seeded into the upper compartment of a 12-well chemotaxis chamber (Neuroprobe, Gaithersburg, MD). Both the upper and lower compartments were filled with α-MEM containing 0.35% BSA and were physically separated by a polycarbonate membrane (8-μm pore size) precoated for 4 h with 100 μg/ml collagen I. Cells were incubated for 36 h at 37°C in 5% CO_2_ humidified conditions, fixed with 4% paraformaldehyde, and stained with 1% borax and 1% methylene blue. Cells of the upper surface of the filter were removed with a cotton swab and those underneath were quantified [29].

### Wound healing assay

MTLn3 cells were grown on a collagen I-precoated 6-well tissue culture plate to about 80% confluency. Cultures were wounded by a heat polished glass pipette (~ 30-50-μm tip) and overlayered with dimethyl polysiloxane (Sigma Aldrich) to reduce evaporation while allowing gas exchange. Detailed observation on the behavior of live cells was monitored by acquiring images every 10 min over a period of 6 h. The effects of ADF or cofilin silencing were assessed by measuring the time and the distance migrated by cells to close the wound. Live cell migration in wound-healing assay was followed using a CCD camera (Motic) on an inverted Leica microscope using 10×, 1.0 NA air objectives.

### Data analysis

Statistical analysis was performed using STATISITCA 7 analysis program (StatSoft Inc., Ok, USA). To determine differences between 3 or more means, one-way ANOVA with Fisher’s LSD for multiple comparisons post-tests were performed. Factorial ANOVA for higher orders (2-way or 3-way) was used to test for interactive effects for multiple categorical independent variables. Results are presented as mean ± standard error of the mean (SEM). All statistical analysis was performed at p < 0.05 level of significance.

## Abbreviations

α-MEM: α-Modified eagle’s media; ABPs: Actin binding proteins; AC: ADF/cofilin; ADF: Actin depolymerizing factor; BSA: Bovine serum albumin; CBS: Cytoskeleton buffer with sucrose; Cfl-1: Cofilin-1; Cfl-2: Cofilin-2; ECM: Extracellular matrix; EGF: Epidermal growth factor; EGFR: Epidermal growth factor receptor; F-actin: Actin filaments; FBS: Fetal bovine serum; KD: Knockdown; MOI: Multiplicity of infection; PBS: Phosphate-buffered saline; PBST: Phosphate-buffered saline containing 0.05% Tween-20; PI3K: Phosphatidylinositol 3-kinase; PIP2: Phosphatidylinositol 4,5-bisphosphate; PLC-γ: Phospholipase γ; siRNA: Small interference RNA; SSH: Slingshot; TAMs: Tumor associated macrophages; TBS: Tris-buffered saline; UB: Ureteric bud; XAC: *Xenopus* ADF/cofilin.

## Competing interests

The authors declare that they have no competing interests.

## Authors’ contributions

LHT carried out immunoassays and microscopy and migration assay, and participated in the design of the study and performed the statistical analysis and helped to draft the manuscript. AES carried out the molecular studies and constructed the adenoviruses. MHH performed the wound healing assay and adhesion assay. SRY participated in the design of the study and coordination. JRB conceived of the study, and participated in its design and coordination and helped to draft the manuscript. All authors read and approved the final manuscript.

## References

[B1] Maeda YT Inose J Matsuo MY Iwaya S Sano M Ordered patterns of cell shape and orientational correlation during spontaneous cell migration PLoS One 2008 3 e3734 10.1371/journal.pone.0003734 19011688 PMC2581918

[B2] Petrie RJ Doyle AD Yamada KM Random versus directionally persistent cell migration Nat Rev Mol Cell Biol 2009 10 538 549 10.1038/nrm2729 19603038 PMC2752299

[B3] Ananthakrishnan R Ehrlicher A The forces behind cell movement Int J Biol Sci 2007 3 303 317 17589565 10.7150/ijbs.3.303 PMC1893118

[B4] Streuli C Pelengaris SM, Khan M Cell Matrix Adhesion, Cell-Cell Interactions, and Malignancy The Molecular Biology of Cancer 2006 3 Oxford: Blackwell Publishing 357 389

[B5] Yamaguchi H Condeelis J Regulation of the actin cytoskeleton in cancer cell migration and invasion: a review Biochim Biophys ACTA 2007 1773 642 652 10.1016/j.bbamcr.2006.07.001 16926057 PMC4266238

[B6] Le Clainche C Carlier M Regulation of actin assembly associated with protrusion and adhesion in cell migration Physiol Rev 2008 88 489 513 10.1152/physrev.00021.2007 18391171

[B7] Saarikangas J Zhao H Lappalainen P Regulation of the actin cytoskeleton-plasma membrane interplay by phosphoinositides Physiol Rev 2010 90 259 289 10.1152/physrev.00036.2009 20086078

[B8] Maciver S Hussey P The ADF/Cofilin family: actin-remodeling proteins: a review Genome Biol 2002 5 3007.1 3007.12 10.1186/gb-2002-3-5-reviews3007 PMC139363 12049672

[B9] Bernstein B Bamburg J ADF/Cofilin: a functional node in cell biology: a review Trends Cell Biol 2010 20 187 195 10.1016/j.tcb.2010.01.001 20133134 PMC2849908

[B10] Kuure S Cebrian C Machingo Q Lu BC Chi X Hyink D D’Agati V Gurniak C Witke W Costantini F Actin depolymerizing factors cofilin1 and destrin are required for ureteric bud branching morphogenesis PLoS Genet 2010 6 e1001176 10.1371/journal.pgen.1001176 21060807 PMC2965756

[B11] Yeoh S Pope B Mannherz H Weeds A Determining the differences in actin binding by human ADF and cofilin J Mol Biol 2002 315 911 925 10.1006/jmbi.2001.5280 11812157

[B12] Andrianantoandro E Pollard TD Mechanism of actin filament turnover by severing and nucleation at different concentrations of ADF/cofilin Mol Cell 2006 24 13 23 10.1016/j.molcel.2006.08.006 17018289

[B13] Ono S Mechanism of depolymerization and severing of actin filaments and its significance in cytoskeletal dynamics Int J Cytol 2007 258 1 82 10.1016/S0074-7696(07)58001-0 17338919

[B14] Chen H Bernstein BW Sneider JM Boyle JA Minamide LS Bamburg JR In vitro activity differences between proteins of the ADF/cofilin family define two distinct subgroups Biochem 2004 43 7127 7142 10.1021/bi049797n 15170350

[B15] Devineni N Minamide LS Niu M Safer D Verma R Bamburg JR Nachmias VT A quantitative analysis of G-actin binding proteins and the G-actin pool in developing chick brain Brain Res 1999 823 129 140 10.1016/S0006-8993(99)01147-6 10095019

[B16] Bernstein B Painter W Chen H Minamide L Abe H Bamburg J Intracellular pH modulation of ADF/Cofilin proteins Cell Mol Biol 2000 47 319 336 10.1002/1097-0169(200012)47:4<319::AID-CM6>3.0.CO;2-I 11093252

[B17] Agnew B Minamide L Bamburg J Reactivation of phosphorylated actin depolymerizing factor and identification of the regulatory site J Biol Chem 1995 270 17582 17587 10.1074/jbc.270.29.17582 7615564

[B18] Niwa R Nagata-Ohashi K Takeichi M Mizuno K Uemura T Control of actin reorganization by slingshot, a family of phosphatases that dephosphorylate ADF/cofilin Cell 2002 108 233 246 10.1016/S0092-8674(01)00638-9 11832213

[B19] Gohla A Birkenfeld J Bokoch G Chronophin, a novel HAD-type serine protein phosphatase, regulates cofilin-dependent actin dynamics Nat Cell Biol 2004 7 21 29 15580268 10.1038/ncb1201

[B20] Huang TY DerMardirossian C Bokoch GM Cofilin phosphatases and regulation of actin dynamics Curr Opin Cell Biol 2006 18 26 31 10.1016/j.ceb.2005.11.005 16337782

[B21] Yonezawa N Nishida E Sakai H pH control of actin polymerization by cofilin J Biol Chem 1985 260 14410 14412 4055781

[B22] Hawkins M Pope B Maciver SK Weeds AG Human actin depolymerizing factor mediates a pH-sensitive destruction of actin filaments Biochem 1993 32 9985 9993 10.1021/bi00089a014 8399167

[B23] Hayden SM Miller PS Brauweiler A Bamburg JR Analysis of the interactions of actin depolymerizing factor with G- and F-actin Biochem 1993 32 9994 10004 10.1021/bi00089a015 8399168

[B24] Pope BJ Zierler-Gould KM Kühne R Weeds AG Ball LJ Solution structure of human cofilin: actin binding, pH sensitivity, and relationship to actin-depolymerizing factor J Biol Chem 2004 279 4840 4848 14627701 10.1074/jbc.M310148200

[B25] Hotulainen P Paunola E Vartiainen M Lappalainen P Actin-depolymerizing factor and cofilin-1 play overlapping roles in promoting rapid F-actin depolymerization in mammalian nonmuscle cells Mol Biol Cell 2005 16 649 664 15548599 10.1091/mbc.E04-07-0555 PMC545901

[B26] Gurniak C Emerald P Witke W The actin depolymerizing factor n-Cofilin is essential for neural tube morphogenesis and neural crest cell migration Dev Biol 2005 278 231 241 10.1016/j.ydbio.2004.11.010 15649475

[B27] Ikeda S Cunningham L Boggess D Hobson C Sundberg J Naggert J Smith R Nishina P Aberrant actin cytoskeleton leads to accelerated proliferation of corneal epithelial cells in mice deficient for destrin (actin depolymerizing factor) Hum Mol Genet 2003 12 1029 1036 10.1093/hmg/ddg112 12700171

[B28] Wiggan O Shaw AE DeLuca JG Bamburg JR ADF/cofilin regulates actomyosin assembly through competitive inhibition of myosin II binding to F-actin Dev Cell 2012 22 530 543 10.1016/j.devcel.2011.12.026 22421043 PMC3306597

[B29] Estornes Y Gay F Gevrey JC Navoizat S Nejjari M Scoazec JY Chayvialle JA Saurin JC Abello J Differential involvement of destrin and Cofilin-1 in the control of invasive properties of Isreco1 human colon cancer cells Int J Cancer 2007 121 2162 2171 10.1002/ijc.22911 17583572

[B30] Nebl G Meuer SC Samstag Y Dephosphorylation of serine 3 regulates nuclear translocation of cofilin J Biol Chem 1996 271 26276 26280 10.1074/jbc.271.42.26276 8824278

[B31] Turhani D Krapfenbauer K Thurnher D Langen H Fountoulakis M Identification of differentially expressed, tumor-associated proteins in oral squamous cell carcinoma by proteomic analysis Electrophoresis 2006 27 1417 1423 10.1002/elps.200500510 16568407

[B32] Unwin RD Craven RA Harnden P Hanrahan S Totty N Knowles M Eardley I Selby PJ Banks RE Proteomic changes in renal cancer and co-ordinate demonstration of both the glycolytic and mitochondrial aspects of the Warburg effect Proteomics 2003 3 1620 1632 10.1002/pmic.200300464 12923786

[B33] Martoglio AM Tom BD Starkey M Corps AN Charnock-Jones DS Smith SK Changes in tumorigenesis- and angiogenesis-related gene transcript abundance profiles in ovarian cancer detected by tailored high density cDNA arrays Mol Med 2000 6 750 765 11071270 PMC1949983

[B34] Aizawa H Sutoh K Tsubuki S Kawashima S Ishii A Yahara I Identification, characterization, and intracellular distribution of cofilin in *Dictyostelium discoideum* J Biol Chem 1995 270 10923 10932 10.1074/jbc.270.18.10923 7738034

[B35] Yap CT Simpson TI Pratt T Price DJ Maciver SK The motility of glioblastoma tumor cells is modulated by intracellular cofilin expression in a concentration-dependent manner Cell Motil Cytoskeleton 2005 60 153 165 10.1002/cm.20053 15662725

[B36] Dang D Bamburg JR Ramos DM Alphavbeta3 integrin and cofilin modulate K1735 melanoma cell invasion Exp Cell Res 2006 312 468 477 10.1016/j.yexcr.2005.11.011 16337627

[B37] Yoshioka K Foletta V Bernard O Itoh K A role for LIM kinase in cancer invasion Proc Natl Acad Sci USA 2003 100 7247 7252 10.1073/pnas.1232344100 12777619 PMC165861

[B38] Bagheri-Yarmand R Mazumdar A Sahin A Kumar R LIM kinase 1 increases tumor metastasis of human breast cancer cells via regulation of the urokinase-type plasminogen activator system Int J Cancer 2006 118 2703 2710 10.1002/ijc.21650 16381000

[B39] Suyama E Wadhwa R Kawasaki H Yaguchi T Kaul SC Nakajima M Taira K LIM kinase-2 targeting as a possible anti-metastasis therapy J Gene Med 2004 6 357 363 10.1002/jgm.491 15026997

[B40] Vlecken DH Bagowski CP LIMK1 and LIMK2 are important for metastatic behavior and tumor cell-induced angiogenesis of pancreatic cancer cells Zebrafish 2009 6 433 439 10.1089/zeb.2009.0602 20047470

[B41] Condeelis J Pollard JW Macrophages: obligate partners for tumor cell migration, invasion, and metastasis Cell 2006 124 263 266 10.1016/j.cell.2006.01.007 16439202

[B42] Mouneimne G Soon L DesMarais V Sidani M Song X Yip S Ghosh M Eddy R Backer J Condeelis J Phospholipase C and cofilin are required for carcinoma cell directionality in response to EGF stimulation J Cell Biol 2004 166 697 708 10.1083/jcb.200405156 15337778 PMC2172433

[B43] vanRheenen J Song X Roosmalen W Cammer M Chen X DesMarais V Yip S Backer J Eddy R Condeelis J EGF-induced PIP_2_ hydrolysis releases and activates cofilin locally in carcinoma cells J Cell Biol 2007 179 1247 1259 10.1083/jcb.200706206 18086920 PMC2140025

[B44] Chen P Xie H Sekar MC Gupta K Wells A Epidermal growth factor receptor-mediated cell motility: phospholipase C activity is required, but mitogen-activated protein kinase activity is not sufficient for induced cell movement J Cell Biol 1994 127 847 857 10.1083/jcb.127.3.847 7962064 PMC2120228

[B45] Chen L Janetopoulos C Huang Y Iijima M Borleis J Devreotes P Two phases of actin polymerization display different dependencies on PI(3,4,5)P3 accumulation and have unique roles during chemotaxis Mol Biol Cell 2003 14 5028 5037 10.1091/mbc.E03-05-0339 14595116 PMC284804

[B46] Chan Y Bailly M Zebda N Segall J Condeelis J Role of cofilin in epidermal growth factor-stimulated actin polymerization and lamellipod protrusion J Cell Biol 2000 148 531 542 10.1083/jcb.148.3.531 10662778 PMC2174812

[B47] Yamaguchi H Yoshida S Muroi E Kawamura M Kouchi Z Nakamura Y Sakai R Fukami K Phosaphatidylinositol 4,5-biphosphate and PIP5-kinaseIα are required for invadopodia formation in human breast cancer cells Cancer Sci 2010 101 1632 1638 10.1111/j.1349-7006.2010.01574.x 20426790 PMC11158062

[B48] Shaw AE Minamide LS Bill CL Funk JD Maiti S Bamburg JR Cross-reactivity of antibodies to actin- depolymerizing factor/cofilin family proteins and identification of the major epitope recognized by a mammalian actin-depolymerizing factor/cofilin antibody Electrophoresis 2004 25 2611 2620 10.1002/elps.200406017 15300782

[B49] Tammana T Sahasrabuddhe A Bajpai V Gupta M ADF/Cofilin-driven actin dynamics in early events of Leishmania cell division J Cell Sci 2010 123 1894 1901 10.1242/jcs.068494 20460437

[B50] Sidani M Wessels D Mouneimne G Ghosh M Goswami S Sarmiento C Wang W Kuhl S El-Sibai M Backer J Eddy R Soll D Condeelis J Cofilin determines the migration behavior and turning frequency of metastatic cancer cells J Cell Biol 2007 179 777 791 10.1083/jcb.200707009 18025308 PMC2080932

[B51] Zebda N Bernard O Bailly M Welti S Lawrence D Condeelis J Phosphorylation of ADF/Cofilin abolishes EGF-induced actin nucleation at the leading edge and subsequent lamellipod extension J Cell Biol 2000 151 1119 1128 10.1083/jcb.151.5.1119 11086013 PMC2174362

[B52] Artym VV Yamada KM Mueller SC ECM degradation assays for analyzing local cell invasion Methods Mol Biol 2009 522 211 219 10.1007/978-1-59745-413-1_15 19247615

[B53] Montanez E Piwko-Czuchra A Bauer M Li S Yurchenco P Fassler R Cheresh DA Integrin Methods of Enzymology 2007 2 San Diego: Academic Press 239 286 10.1016/S0076-6879(07)26012-4 17697888

[B54] Wang W Goswami S Lapidus K Wells AL Wyckoff JB Sahai E Singer RH Segall JE Condeelis JS Identification and testing of a gene expression signature of invasive carcinoma cells within primary mammary tumors Cancer Res 2004 64 8585 8594 10.1158/0008-5472.CAN-04-1136 15574765

[B55] Wang W Mouneimne G Sidani M Wyckoff J Chen X Makris A Goswami S Bresnick AR Condeelis JS The activity status of cofilin is directly related to invasion, intravasation, and metastasis of mammary tumors J Cell Biol 2006 173 395 404 10.1083/jcb.200510115 16651380 PMC2063840

[B56] Zaidel-Bar R Cohen M Addadi L Geiger B Hierarchical assembly of cell matrix adhesion complexes Biochem Soc T 2004 32 416 420 10.1042/BST0320416 15157150

[B57] Hotulainen P Lappalainen P Stress fibers are generated by two distinct actin assembly mechanisms in motile cells J Cell Biol 2006 173 383 394 10.1083/jcb.200511093 16651381 PMC2063839

[B58] Chen C Alonso J Ostuni E Whitesides G Ingberb D Cell shape provides global control of focal adhesion assembly Biochem Biophys Res Comm 2003 307 355 361 10.1016/S0006-291X(03)01165-3 12859964

[B59] Deguchi S Sato M Biomechanical properties of actin stress fibers of non-motile cells Biorheology 2009 46 93 105 19458413 10.3233/BIR-2009-0528

[B60] Guo WH Wang YL A three component mechanism for fibroblast migration with a contractile cell body that couples a myosin II-independent propulsive anterior to a myosin II-dependent resistive tail Mol Biol Cell 2012 23 1657 1663 10.1091/mbc.E11-06-0556 22398722 PMC3338433

[B61] Horita Y Ohashi K Mukai M Inoue M Mizuno K Suppression of the invasive capacity of rat ascites hepatoma cells by knockdown of slingshot or LIM-kinase J Biol Chem 2008 283 6013 6021 10.1074/jbc.M706538200 18171679

[B62] Toshima J Toshima J Amano T Yang N Narumiya S Mizuno K Cofilin phosphorylation by protein kinase testicular protein kinase 1 and its role in integrin-mediated actin reorganization and focal adhesion formation Mol Biol Cell 2001 12 1131 1145 10.1091/mbc.12.4.1131 11294912 PMC32292

[B63] Marshall TW Aloor HL Bear JE Coronin 2A regulates a subset of focal-adhesion-turnover events through the cofilin pathway J Cell Sci 2009 122 3061 3069 10.1242/jcs.051482 19654210 PMC2729258

[B64] Mseka T Bamburg J Cramer L ADF/Cofilin family proteins control formation of oriented actin-filament bundles in the cell body to trigger fibroblast polarization J Cell Sci 2007 120 4332 4344 10.1242/jcs.017640 18042624

[B65] Dawe H Minamide L Bamburg J Cramer L ADF/Cofilin controls cell polarity during fibroblast migration Curr Biol 2003 13 252 257 10.1016/S0960-9822(03)00040-X 12573223

[B66] Mseka T Cramer LP Actin depolymerization-based force retracts the cell rear in polarizing and migrating cells Curr Biol 2011 21 2085 2091 10.1016/j.cub.2011.11.006 22137472

[B67] Vartiainen K Mustonen T Mattila K Ojala J Thesleff I Partanen J Lappalainen P The three mouse actin-depolymerizing factor/cofilins evolved to fulfill cell-type-specific requirements for actin dynamics Mol Biol Cell 2002 13 183 194 10.1091/mbc.01-07-0331 11809832 PMC65081

[B68] Lee CW Vitriol EA Shim S Wise AL Velayutham RP Zheng HQ Dynamic localization of G-actin during membrane protrusion in neuronal motility Curr Biol 2013 23 1 11 23746641 10.1016/j.cub.2013.04.057 PMC3712510

[B69] Rochelle T Daubon T Van Troys M Harnois T Waterschoot D Ampe C Roy L Bourmeyster N Constantin B p210bcr-abl induces amoeboid motility by recruiting ADF/destrin through RhoA/ROCK1 FASEB J 2013 27 123 134 10.1096/fj.12-205112 23047898

[B70] Brummelkamp TR Bernards R Agami R Stable suppression of tumorigenicity by virus-mediated RNA interference Cancer Cell 2002 2 243 247 10.1016/S1535-6108(02)00122-8 12242156

[B71] He TC Zhou S da Costa LT Yu J Kinzler KW Vogelstein B A simplified system for generating recombinant adenoviruses Proc Natl Acad Sci USA 1998 95 2509 2514 10.1073/pnas.95.5.2509 9482916 PMC19394

[B72] Garvalov B Flynn K Neukirchen D Meyn L Teusch N Wu X Brakebusch C Bamburg J Bradke F Cdc42 regulates cofilin during the establishment of neuronal polarity J Neurosci 2007 27 13117 13129 10.1523/JNEUROSCI.3322-07.2007 18045906 PMC6673401

[B73] Minamide L Shaw A Sarmiere P Wiggan O Maloney M Bernstein B Sneider J Gonzalez J Bamburg J Production and use of replication-deficient adenovirus for transgene expression in neurons Methods Cell Biol 2003 71 387 416 12884701 10.1016/s0091-679x(03)01019-7

[B74] Wessel D Flügge UI A method for the quantitative recovery of protein in dilute solution in the presence of detergents and lipids Anal Biochem 1984 138 141 143 10.1016/0003-2697(84)90782-6 6731838

